# Estimating size specific dose estimate from computed tomography radiograph localizer with radiation risk assessment

**DOI:** 10.1002/acm2.13989

**Published:** 2023-05-03

**Authors:** Christiane Sarah Burton, Shahad Al‐Ward

**Affiliations:** ^1^ Department of Diagnostic Imaging St Jude Children's Research Hospital Memphis Tennessee USA; ^2^ Department of Radiation Oncology St Jude Children's Research Hospital Memphis Tennessee USA

**Keywords:** CT, localizer, SSDE, water‐equivalent diameter

## Abstract

**Background:**

Quantifying radiation burden is necessary for optimizing imaging protocols. The normalized dose coefficient (NDC) is determined from the water‐equivalent diameter (WED) and is used to scale the CTDIvol based on body habitus to determine the size specific dose estimate (SSDE). In this study we determine the SSDE prior to the CT scan and how sensitive the SSDE from WED is to the lifetime attributable risk (LAR) from BEIR VII.

**Method:**

For calibration, phantom images are used to relate the mean pixel values along a profile ($\bar{\mathrm{PPV}}$) of the CT localizer to the water‐equivalent area (*A*_W_) of the CT axial scan at the same z‐location. Images of the CTDIvol phantoms (32 cm, 16 cm, and ∼1 cm) and ACR phantom (Gammex 464) were acquired on four scanners. The relationship between the *A*_W_ and $\bar{\mathrm{PPV}}$ was used to calculate the WED from the CT localizer for patient scans. A total of 790 CT examinations of the chest and abdominopelvic regions were used in this study. The effective diameter (ED) was calculated from the CT localizer. The LAR was calculated based on the patient chest and abdomen using the National Cancer Institute Dosimetry System for Computed Tomography (NCICT). The radiation sensitivity index (RSI) and risk differentiability index (RDI) were calculated for SSDE and CTDIvol.

**Results:**

The WED from CT localizers and CT axials scans show good correlation (*R* ^2^ = 0.96) with the maximum percentage difference being 13.45%. The NDC from WED correlates poorly with LAR for lungs (*R* ^2^ = 0.18) and stomach (*R* ^2^ = 0.19), however that is the best correlation.

**Conclusion:**

The SSDE can be determined within 20% as recommended by the report of AAPM TG 220. The CTDIvol and SSDE are not good surrogates for radiation risk, however the sensitivity for SSDE improves when using WED instead of ED.

## 1 INTRODUCTION

The medical imaging community has taken initiative to reduce dose from computed tomography (CT). As of right now, vendors are starting to migrate from using the CT Dose Index (CTDI) towards using the size specific dose estimate (SSDE). Currently it is possible to measure the SSDE using geometric size surrogates, lateral (LAT) and anterior‐posterior (AP) dimensions, and effective diameter (ED) from the CT localizer. However, due to difference in attenuation of lung tissue,^1^ having an estimate of the water‐equivalent diameter (WED), which considers patient attenuation information, from the CT localizer prior to the CT scan would be useful. There is also a question of whether SSDE using the WED is a better metric of lifetime attributable risk (LAR) to a patient compared to SSDE calculated from LAT and AP, and just the CTDI_vol_ alone.

The CTDI_vol_ only reflects the system's radiation output^1^, ^2^, ^3^, ^4^ for a specific set of conditions in a cylindrical acrylic polymethyl methacrylate (PMMA) phantom with either 16 cm or 32 cm diameters with a contiguous axial or helical examination.^4^ As a result, using data from previous studies,^5^, ^6^, ^7^, ^8^ the American Association of Physicists in Medicine (AAPM) Task Group (TG) 220 introduced the water equivalent diameter (WED) for estimating patient size in computed tomography (CT). The size specific dose estimate (SSDE) was introduced to represent the absorbed dose to the patient and it is calculated using a scaling factor known as the normalized dose coefficient (NDC) for the CTDI_vol_ based on these patient size surrogates. According to the AAPM TG Report 220, using the water‐equivalent diameter (WED) is the “preferred” method in determining the SSDE because it factors in patient attenuation properties. Burton and Szczykutowicz^9^ demonstrated that calculating patient size surrogates such as WED from patient data was similar to the results of the AAPM TG Reports 204 and 220,^10^ thus providing the medical imaging community with a gold standard for calculating WED from patient data. Zhang et al. determined a calibration method that may be used to determine the WED from CT localizers using phantoms,^11^ however it has not been extended to patient scans.

**Main points**

‐ The water‐equivalent diameter can be accurately determined from the CT localizer radiographs for patient scans to well within 20% as recommended by the AAPM TG Report 220.
‐ Estimating the water‐equivalent diameter from CT localizer radiographs would allow it to be included into data‐driven clinical workflows such as size adaptive protocol selection using diagnostic reference ranges which provide a minimum estimated patient dose.
‐ The size specific dose estimate (SSDE) for lungs and abdomen correlates better with lifetime attributable risk compared to SSDE calculated from effective diameter and CTDIvol. The radiation sensitivity improves with SSDE calculated from water‐equivalent diameter (WED) compared to using the effective diameter. There is a loss in sensitivity to radiation using SSDE to CTDIvol. Neither the SSDE nor CTDIvol are good metrics for risk surrogates.

The metric for LAR in BEIR VII^12^ considers characteristics of the patient including sex, age, and tissue. There are few studies that look at risk to patient populations in diagnostic procedures because it is cumbersome to calculate. Dose metrics such as SSDE and CTDI_vol_ are sometimes used to assess risk to patients. It was reported that the SSDE is less sensitive to radiation risk compared to CTDI_vol_,^13^ however it was unclear whether the WED was used to calculate the SSDE. The chest region will result in a different estimate of patient size surrogate between ED and WED. To understand if SSDE calculated from WED would be a better surrogate to quantify risk compared to SSDE calculated from ED, and CTDI_vol_ alone, it needs be measured against the LAR for the same patient.

In this study, the WED from the CT localizers will be compared to the gold standard WED from CT axial scans to demonstrate the accuracy. Furthermore, the LAR from the SSDE using WED and geometric size surrogates and CTDIvol will be compared.

## 2 METHODS AND MATERIALS

**FIGURE 1:**
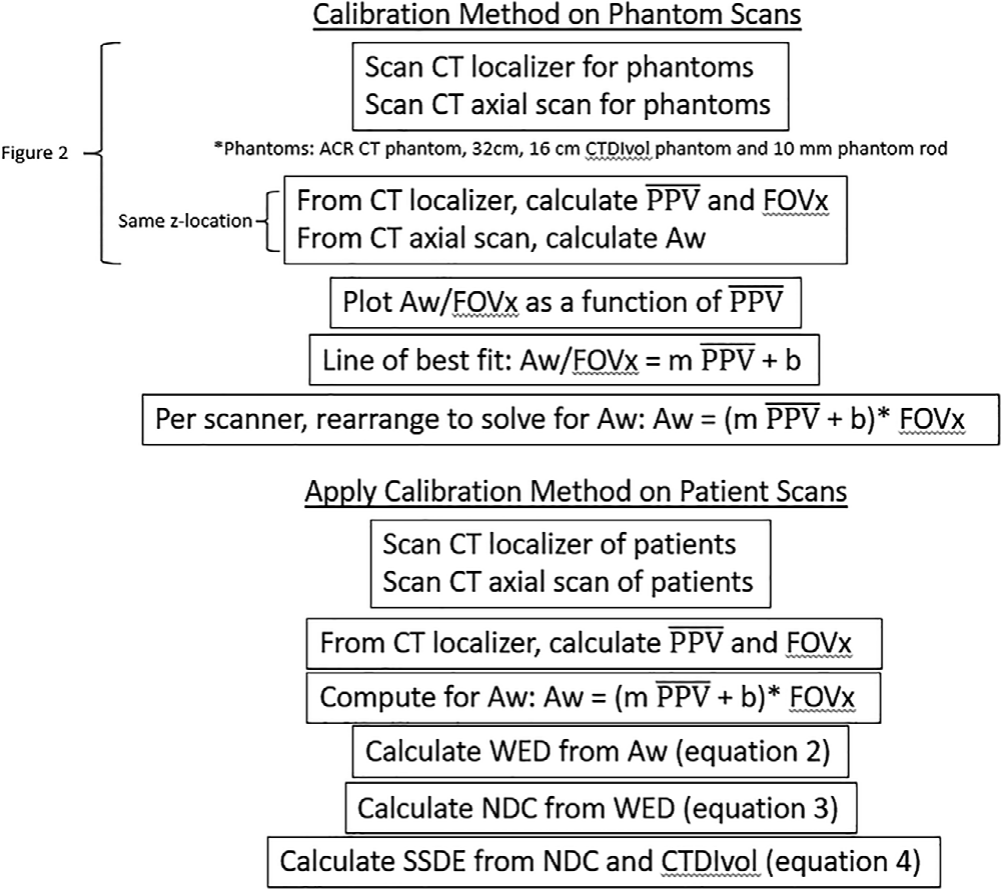
Flow chart for the calibration method on the phantom scans and how the calibration method is applied to the patient scan per scanner. Figure 2 and Equations (2), (3), and 4 are referenced in this flowchart.

Figure 1 provides a flow chart for the calibration method on the phantom scans and how the calibration method is applied to the patient scan to ultimately determine the SSDE prior to the scan from the WED.

### 2.1 Water‐equivalent diameter calculation

The gold standard estimate for *A*_W_ comes from the CT axial images using Equation 3a of the report of TG 220, reproduced here:

(1) $$A_{w}=\sum \left(\frac{ct(x,y)}{1000}+1\right)\times A_{ROI}$$

The CT(x,y) is the CT number and *A*_ROI_ is the total area of the region of interest (ROI). Note that the *A*_W_ depends on the CT number in Hounsfield Units and the area of the ROI therefore whether the automatic exposure control (AEC) is on/off should not affect the computed value.

The WED was determined by using Equation 3d in the report of TG 220, reproduced here:

(2) $$\mathrm{WED}=2\frac{\sqrt{A_{W}}}{p}$$

Relating the $\bar{\mathrm{PPV}}$ to *A*_W_ allows the $\bar{\mathrm{PPV}}$ to be used to calculate WED from the CT localizer. The normalized dose coefficient (NDC) uses the WED to scale the CTDIvol to calculate the SSDE.

### 2.2 Calibration overview

The calibration approach relates the mean profile pixel values ($\bar{\mathrm{PPV}}$) in the CT localizer to the water‐equivalent area (*A*_W_) in the CT axial image at the same z‐location. By setting up a relationship between two quantities, $\bar{\mathrm{PPV}}$ and *A*_w_, that are equivalent for CT localizer and CT axial scans for a single scanner, the *A*_w_ can be reliably determined from the $\bar{\mathrm{PPV}}$ for patient scans. Toth et al. have demonstrated how to measure the total object attenuation is like *A*_w_ of the same z‐location by taking the product of the $\bar{\mathrm{PPV}}$ and field of view in the x‐direction (FOV_X_). The FOV_X_ for the CT localizer is fixed therefore testing the correlation between the total object attenuation in the $\bar{\mathrm{PPV}}$ of the CT localizer and relationship $R=\frac{A_{w}}{FOV_{x}}$. This linear calibration curve is plotted for $\bar{\mathrm{PPV}}$ and *R* for various kVps for CT localizers and CT axial scans using phantoms with water‐equivalent properties. The water‐equivalent diameter (WED) is determined from the *A*_w_ as shown in Equation 2.

Estimating the effective diameter (ED) from the CT localizer does not require a calibration curve. However, it does require a magnification correction outlined in Burton et al.^14^ that will be applied here.

### 2.3 Phantom acquisition

CT localizer and CT axial images 0.625 mm slice thicknesses of the CTDI_vol_ and ACR phantom were acquired on GE Revolution (GE HealthCare, Chicago, IL, USA), SIEMENS Intevo (SIEMENS AG, Munich, Germany), Discovery (GE HealthCare, Chicago, IL, USA), iQon (Koninklijke Philips N.V., Amsterdam, Netherlands), and Philips VEREOS PET/CT (Koninklijke Philips N.V., Amsterdam, Netherlands). The “rod,” otherwise known as the acrylic rod used in the CTDI_vol_ phantom, was used to achieve measurements closest to the zero origin for this calibration plot. Figure 1 shows an example of the profile along the mean profile pixel value $\left(\bar{\mathrm{PPV}}\right)$ along the x‐dimension of the CT localizer and the image manipulated to calculate the water‐equivalent diameter (*A*_W_) in the CT axial image at the same z‐location. These images were read into programming and numeric computing platform (MATLAB, the Mathworks INC, Natick, MA, USA) where the images could be analyzed and the DICOM tag data could be retrieved from the DICOM header. The DICOM tag Image Patient Position (0020,0032) was used to determine where the top corner of the CT localizer is along the z‐axis. To determine the CT axial slice locations along the z‐axis, the minimum and maximum slice location were found using DICOM tag Slice Location (0020,1041) and were subtracted from the z‐location of the top corner of the CT localizer. The minimum and maximum slice locations were divided by the pixel spacing from the CT localizer using DICOM tag Pixel Spacing (0028,0030). The width of the field of view along the x‐direction (FOV_X_) was taken as the product of the number of columns using MATLAB command size with option 2 and pixel size using DICOM tag Pixel Spacing (0028,0030).

**FIGURE 2:**
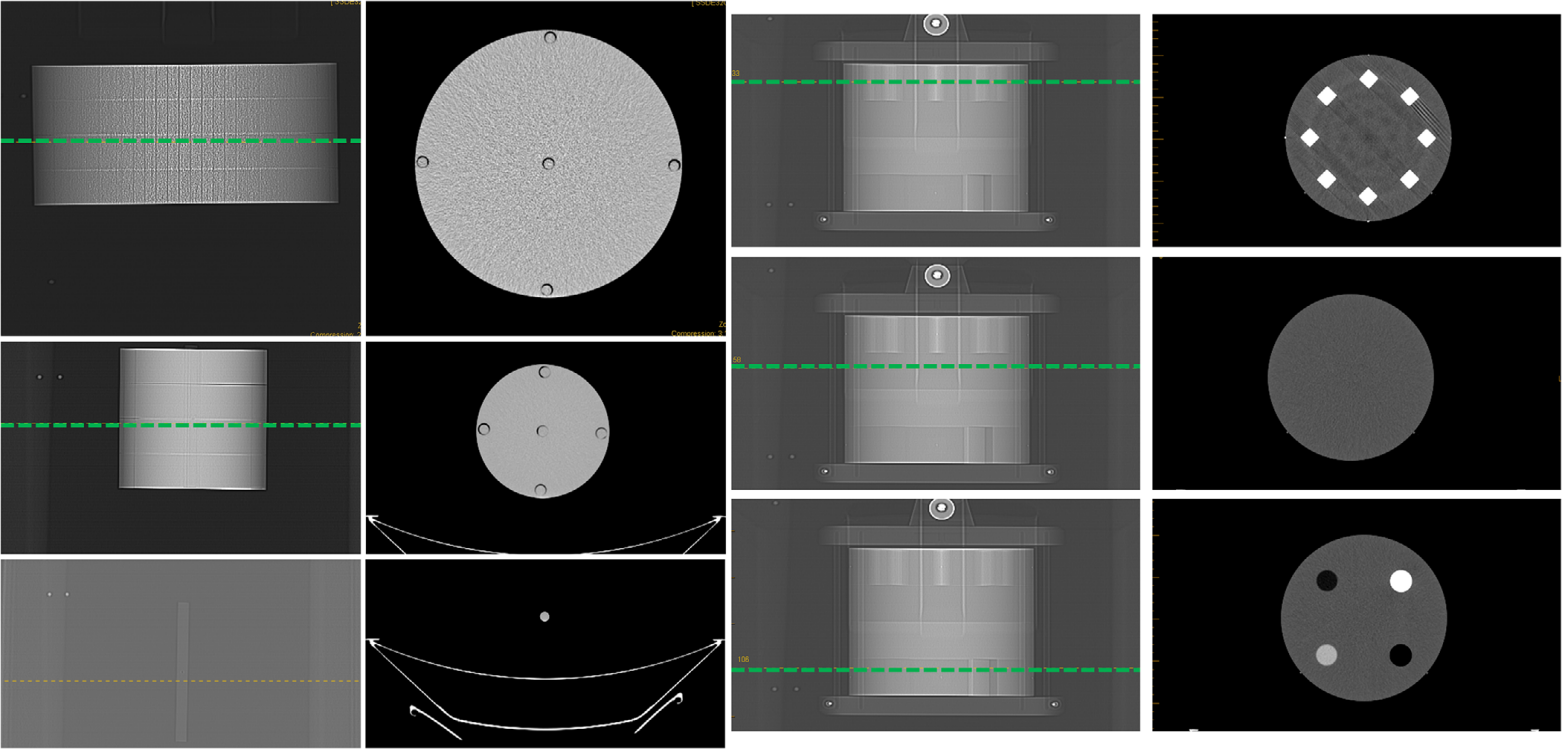
shows (a) the correlation between the CT localizer‐based WED as a function of CT axial‐based WED (*R* ^2^ = 0.96, 95% confidence interval of 16 mm) with VEREOS (*R* ^2^ = 0.99), iQon (*R* ^2^ = 0.99), Siemens (*R* ^2^ = 0.96), and GE Revolution (*R* ^2^ = 0.96) and (b) the correlation between the CT localizer‐based NDC as a function of CT axial‐based NDC for all scanners.

Figure 2 shows visual of the ACR (right), 32 cm CTDIvol (top left), 16 cm CTDIvol (middle left) and acrylic rod (bottom left) phantoms examples of profiles along the row of the CT localizer used for the mean profile pixel value ($\bar{\mathrm{PPV}}$), and corresponding CT axial images at the same z‐location used calculate water‐equivalent area (*A*_W_) with the CT numbers taken within the area of the region of interest (*A*_ROI_).

### 2.4 Calibration curves

**FIGURE 3:**
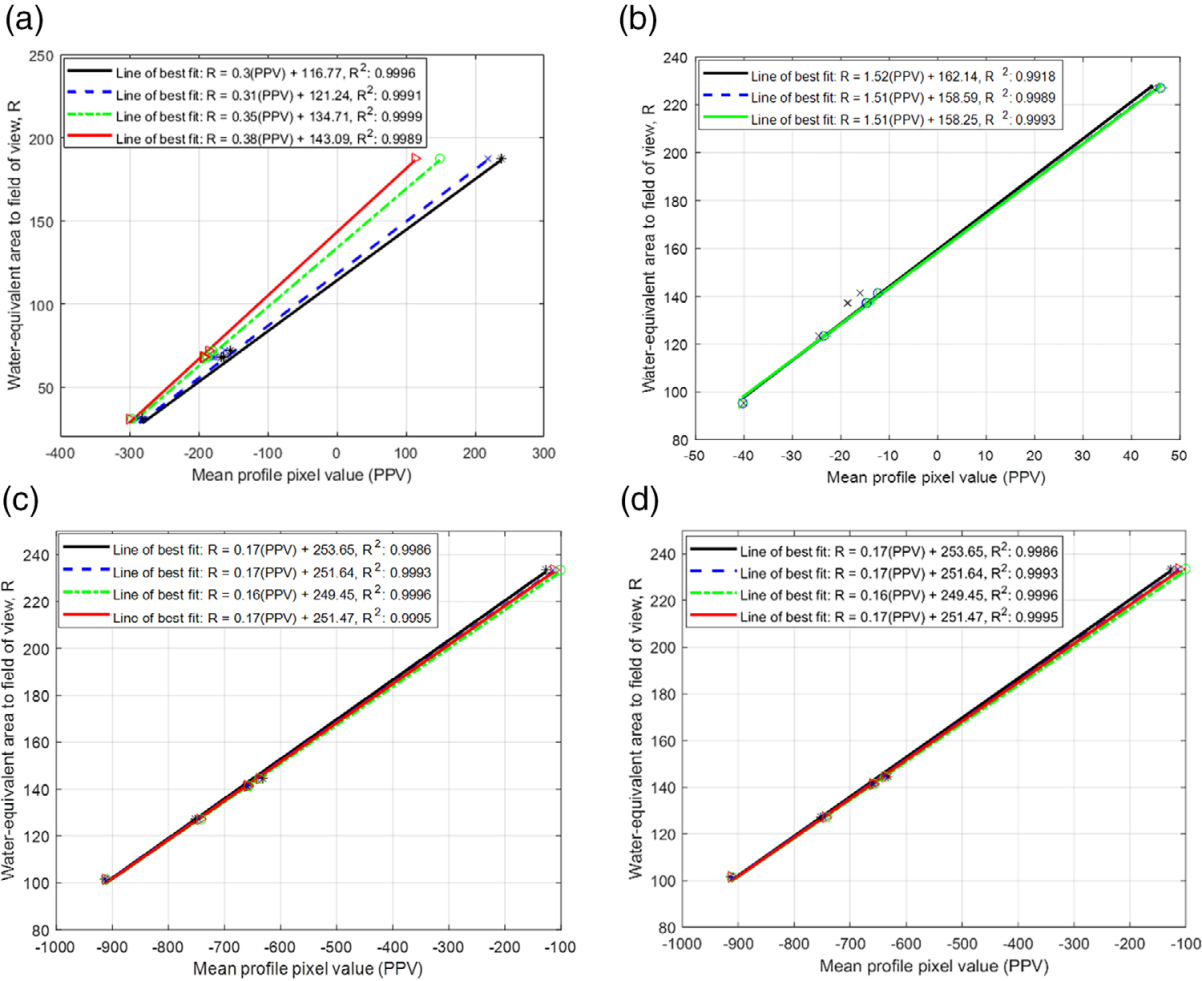
Example of calibration curves for (a) GE Revolution with CT axial scans of phantoms acquired at 120 kV and CT localizers acquired at 70, 80, 100, and 120 kV with *R* ^2^ > 0.99, (b) SIEMENS Intevo with CT axial scans acquired at 110 kV and CT localizers acquired at 80, 110, and 130 kV with *R* ^2^ > 0.99, (c) Philips iQon with CT axial scans at 120 kV and CT localizers acquired at 80, 100, 120, and 140 kV, and (d) Philips VEREOS PET/CT with CT axial scans at 120 kV and CT localizers acquired at 80, 100, 120, and 140 kV.

Figure 3a‐d shows examples of calibration linear relationships generated for the GE Revolution, SIEMENS Intevo, Philips iQon and Philips VEREOS PET/CT using the different phantom sizes. The water‐equivalent area (*A*_W_) normalized to the field of view in the x‐dimension of the CT localizer (FOV_X_), denoted as *R*, as a function of the mean profile pixel value ($\bar{\mathrm{PPV}}$) for the most common kVp for axial scans on each scanner. The lines of best fit (LBF) for these linear plots are used to convert the $\bar{\mathrm{PPV}}$ to *A*_W_. Figure 1 shows steps for the calibration method and how it is applied to patient data in the next section.

### 2.5 Patient data collection

This study was performed in compliance with the Health Insurance Portability and Accountability Act (HIPAA) and all data were collected under an institutional review board (IRB)‐approved protocol in a retrospective way where the patient consent was waived. The data are a mix of adult and pediatric patients (above and below the age of 18). The CT localizer and CT axial images of patients were collected on all four scanners. Table 1 provides some key information about the pediatric data collected. The phantom data were collected to provide a calibration method to relate the pixel value in the CT localizer of the patient to the water‐equivalent area *A*_w_ so the WED may be calculated.

**TABLE 1:** Experimental data collection of human patient routine cases performed on the GE Revolution, SIEMENS Intevo, Philips iQon, and Philips VEREOS PET/CT for the CT scans for pediatric patients.

Manufacturer	Model	Software version	Number of patients	kV	Kernel Recon CT axial	Kernel Recon CT localizer
GE	Revolution	revo_ct_22b14_sp.3	184	120	STND	STND
SIEMENS	Intevo	VB10B	207	80	B08s	T80f
PHILIPS	iQon	4.7	202	120	STANDARD	STANDARD
PHILIPS	VEREOS	4.1	197	120	STANDARD	STANDARD

Experimental data collection of human patient routine cases performed on the GE Revolution and SIEMENS Intevo for the CT scans for pediatric (Table 1).

**FIGURE 4:**
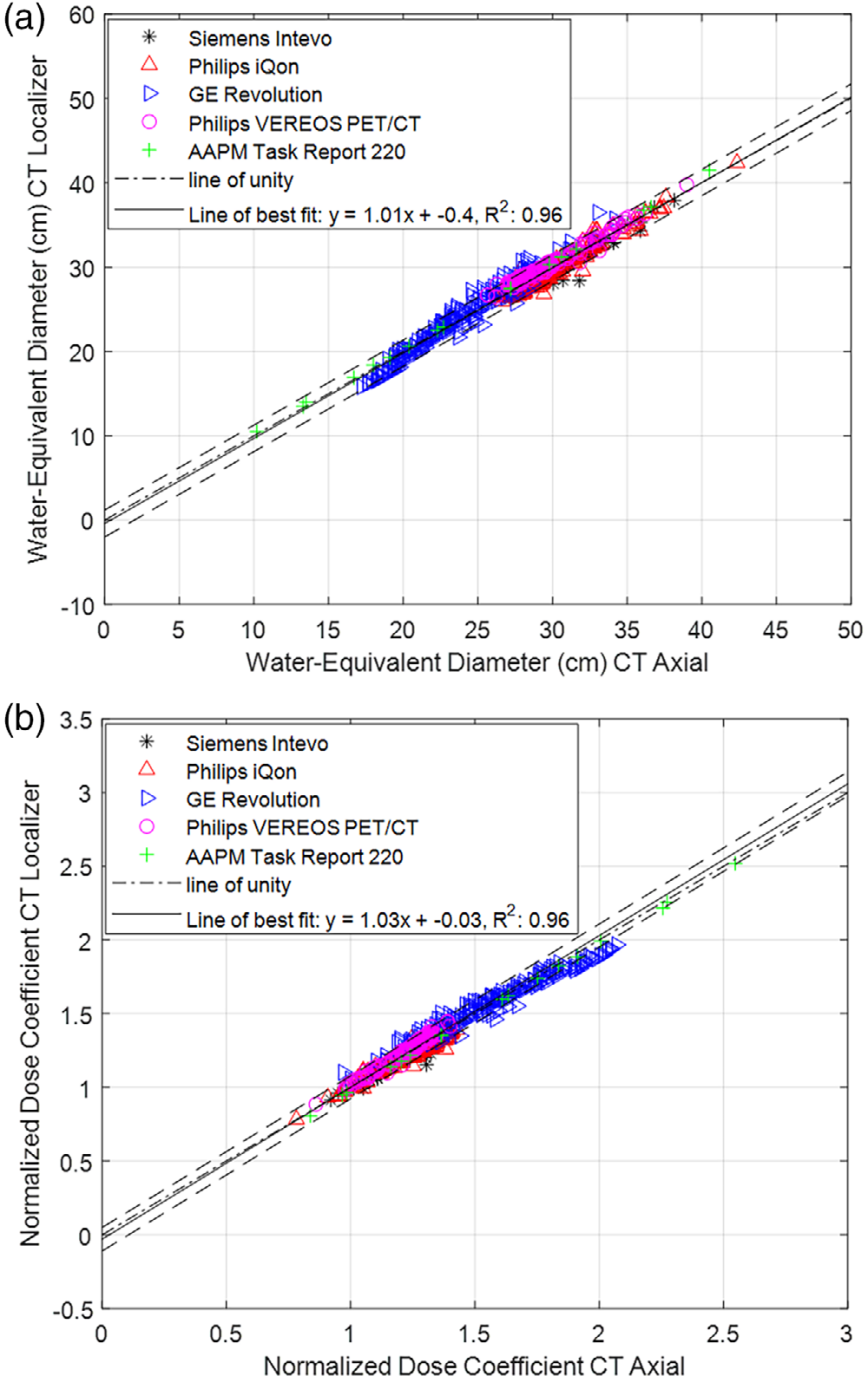
(a) Water‐equivalent diameter (WED) estimated from CT localizer as a function of CT axial‐based water‐equivalent diameter for GE Revolution, SIEMENS Intevo, Philips iQon, and VEREOS PET/CT. (b) Normalized dose coefficient (NDC) calculated from CT localizer WED as a function of NDC calculated from CT axial‐based WED for GE Revolution, SIEMENS Intevo, Philips iQon, and VEREOS PET/CT. The line of best fit and 95% confidence interval (CI) are plotted for all graphs, including the results from the report of TG 220. The *R* ^2^ value represents the correlation for the entire data set.

Using the CT localizer, the water‐equivalent area, *A*_W_, is obtained using the calibration equations which are the line‐of‐best‐fit (LBF) on each of the calibration plots. For comparison, the WED was calculated for each CT axial slice along the scan length and averaged over the entire scan length. The report of TG 204 provides the equation for normalized dose coefficient (NDC) that use the WED replicated here as Equation 3 (REF: Equation A‐1 and coefficients from Figure 4 in report).^6^

(3) NDC=a×exp(−b×D)

where constants *a* = 3.70469 and *b* = 0.03671937 found in the AAPM TG Report 204 for a 32 cm PMMA phantom, the *D* represents the water‐equivalent diameter (WED) or effective diameter (ED), and the normalized dose coefficient is denoted as NDC. This equation is applicable to WED and ED for both CT localizer and CT axial scans. The size specific dose estimate (SSDE) is simply taking the product of the CTDIvol and the NDC,

(4) SSDE=NDC×CTDIvol

where the CTDIvol is taken from the patient's dose report.

To assess the NDC calculated from the CT localizers, the CT localizer‐based NDC was compared to the CT axial‐based NDC for all scanners by plotting it. All plotted data were compared to a line of unity, which represents an exact match. The results were compared to the phantom and Monte Carlo results in the report of TG 220. A 95% confidence interval was plotted for CT localizer‐based WED as a function of CT axial‐based WED. Outliers are defined as points outside of the 95% confidence interval and were analyzed.

The risk index (RI) was calculated based on the actual organ dose specific to a patient and the lifetime attributable risk of cancer incidence.^15^ The organ dose to chest (lungs) and abdomen pelvis (stomach) was calculated using the National Cancer Institute Dosimetry System for Computed Tomography (NCICT) which factors in patient sex, height, weight, region of body scanned, age, kVp, and the CTDIvol.^16^ The lifetime attributable risk of cancer incidence in the US population is reported in BEIR VII which considers the gender, age, and type of tissue (i.e., lung and stomach).^12^, ^13^ The SSDE was determined using WED and effective diameter from the CT localizers,^14^ and CTDIvol estimated prior to the scan. A linear regression was determined between the SSDE, CTDIvol, and the RI. The radiation sensitivity index (RSI) was determined by taking the slope and normalizing it to the ratio of mean of the RI to the mean of the SSDE and WED. The closer the RSI is to 1, the better the metric.^15^ The risk differentiability index (RDI) was determined by dividing the root mean square error (RMSE) to the slope to demonstrate how well the radiation risk varies across different procedures.

## 3 RESULTS

### 3.1 WED values

Figure 2a,b shows the relationship between the WED and NDC calculated from the CT localizer and the CT axial scan with most points falling within the 95% confidence interval. Table 2 shows the maximum, mean, and minimum percentage difference between the WED and NDC calculated from the CT localizer and CT axial scan where the maximum difference is 13.17% for WED and 13.45% for NDC. The radiation risk index (RI), radiation sensitivity index (RSI), and risk differentiability index (RDI) are reported for lung and stomach in Tables 3 and 4, respectively.

**TABLE 2:** The absolute maximum, mean, and minimum percentage differences for CT localizer WED to CT axial WED for the GE Revolution, SIEMENS Intevo, Philips iQon, and Philips VEREOS PET/CT scanners

System	Maximum percentage difference WED (%)	Maximum percentage difference NDC (%)	Mean percentage difference WED (%)	Mean percentage difference NDC(%)	Minimum percentage difference WED (%)	Minimum percentage difference NDC (%)
GE Revolution	13.17	13.45	4.68	3.16	0.00	0.00
SIEMENS Intevo	11.94	11.71	1.96	2.09	0.08	0.10
Philips iQon	9.64	9.07	1.91	2.03	0.01	0.01
Philips VEREOS PET/CT	6.09	6.45	1.96	2.17	0.11	0.13

**TABLE 3:** The linear regression, radiation sensitivity index (RSI) and radiation difference index (RDI) for chest for the Normalized Dose Coefficient (NDC) using the water‐equivalent diameter (*D*_W_) and effective diameter (DE) calculated from water‐equivalent diameter and effective diameter, and CTDIvol

Metric	*R* ^2^	RSI	RDI (number of cancers per 1000 patients per 100 mGy)
NDC WED localizer	0.18	0.062	0.39
SSDE WED localizer	0.051	0.089	0.22
NDC DE localizer	0.039	0.048	0.34
SSDE DE localizer	0.035	0.070	0.27
CTDIvol	0.057	0.13	0.27

**TABLE 4:** The linear regression, radiation sensitivity index (RSI) and radiation difference index (RDI) for abdominopelvis for the Normalized Dose Coefficient (NDC) using the water‐equivalent diameter (*D*_W_) and effective diameter (DE) calculated from water‐equivalent diameter and effective diameter, and CTDIvol

Metric	*R* ^2^	RSI	RDI (number of cancers per 1000 patients per 100 mGy)
NDC WED localizer	0.19	0.076	0.13
SSDE WED localizer	0.012	0.050	0.16
NDC DE localizer	0.17	0.071	0.18
SSDE ED localizer	0.011	0.048	0.19
CTDIvol	0.027	0.11	0.13

Table 2 show the absolute maximum, mean, and minimum percentage difference for WED and NDC.

### 3.2 Radiation risk

Tables 3 and 4 show the lifetime attributable risk (LAR) as determined from BEIR VII as a function of Normalized Dose Coefficients (NDC) as calculated from WED and ED, and the CTDIvol. The LAR has the highest correlation with NDC from WED for chest.

## 4 DISCUSSION

The water‐equivalent diameter (WED) can be determined from CT localizers using the calibration method outlined in this article. The calibration method used in this article was introduced by Zhang et al. as way to estimate the WED from the CT localizer. They did not validate their approach using patient data as we have done in this study, nor did their study look at assessing SSDE as a surrogate for radiation risk. In our study, the correlation between the mean pixel value and water‐equivalent area normalized to the field of view was excellent for all calibration curves. To achieve an accurate estimate of WED, these calibration curves are necessary on every scanner model. They should be generated for every voltage available for CT localizer and CT axial scan. The patient data were filtered for the appropriate kV and the conversion was performed using the line of best fit from the calibration curve for $\bar{\mathrm{PPV}}$ to *A*_W_, thus giving accurate results for WED from CT localizers. The calibration method is vendor and technique‐specific, therefore it would be in the best interest of the vendor to scan phantoms of different sizes once at different kVps to generate multiple curves so that SSDE may be estimated from the patient data prior to each scan. We used three different vendors on four different scanners to demonstrate that these curves would need to be generated for each independent scanner. This is because, unlike CT numbers, the pixel values in the CT localizers are not absolute and these numbers could potentially vary with software version. If properly calibrated, the CT numbers are absolute and will not vary between vendors given that a standard kernel is used—only slight variations will be due to noise.

When comparing WED and NDC between CT localizers and CT axial scans, there was excellent correlation (*R* ^2^ = 0.96) for all scanners with most data points falling within the 95% confidence interval. An accurate estimate of WED from the CT localizers translates to an accurate estimate of the CT localizer based NDC, as shown in Figure 2b. The CT axial scans are considered the gold‐standard for patient size, therefore excellent agreement between the CT localizer‐based WED and NDC would translate into an accurate estimate of SSDE prior to the patient scan.

There were few outlier cases that fall outside of 95% confidence interval that were due to truncation in the CT axial image. It should be noted that the report of TG 220 charges that the implemented method be shown to determine WED to within 20% of the reference value over a range of patient sizes. In Table 3 the GE Revolution shows a maximum percentage difference in 13.17% for WED and 13.5% for NDC, and the average percentage difference is 2.38% and 2.35%, respectively. These were the highest differences reported from all four scanners.

The American Association of Physicists in Medicine (AAPM) Report 204 was the first formal report to propose the concept of SSDE.^5^ The report of TG 204 uses the patient's geometric size surrogates, anterior‐posterior (AP), lateral (LAT), and effective diameter ($\sqrt{AP\times LAT}$), but the geometric size surrogates do not take into account the attenuation properties of various tissue types including the lung which has a much lower density compared to PMMA.^6^ The report of TG 220 addressed this limitation, and this led to concept of water‐equivalent diameter (WED) which considers tissue attenuation in addition to patient geometric size. The report of TG 220 recommends that calculations of SSDE use the WED instead of the effective diameter whenever possible.^7^ The report of TG 220 generated results using phantom experiments and Monte Carlo simulations and phantom data. Using patient data, the results from AAPM TG Reports 204 and 220 were confirmed and that provided the CT imaging community with a gold standard for patient size estimate.^8^ This provided a means to explore estimating patient size surrogates from CT localizers as shown in Figure 2a.

It has been shown that the geometric size surrogates can be determined directly from CT localizers.^12^, ^13^ The magnification method^14^ considered where the x‐rays intersected with an elliptical patient and projected onto the detector. A notable discovery from these studies was that the ACR DIR method is limited to a certain range of patient sizes. For smaller patients, the pixel values for the couch went above the threshold and were, therefore, generally incorporated into the calculation to make the patient seem larger. For bariatric patients, the pixel values for part of the patient went below the threshold and therefore were not included in the estimate of patient size. The effective diameter calculated from the CT localizer could be compared to the CT axial scan.

Recently, Zhang et al. demonstrated a way of estimating WED from CT localizers using a calibration between localizer pixel values (LPV) and attenuation that requires one CT localizer.^9^ The calibration was demonstrated using phantom data (CTDI, ACR phantoms) where the methodology of the report of TG 220 was used to relate the water‐equivalent length from the CT localizer to water‐equivalent area. What is unknown from this study is whether this calibration is vendor specific and technique specific. Typically, CT scanners come with tube potentials that range from 80 to 140 kV. The pixel values from the CT localizers are not absolute and therefore are kV dependent which will affect the calibration curves as shown in Figure 1a‐d.

**FIGURE 5:**
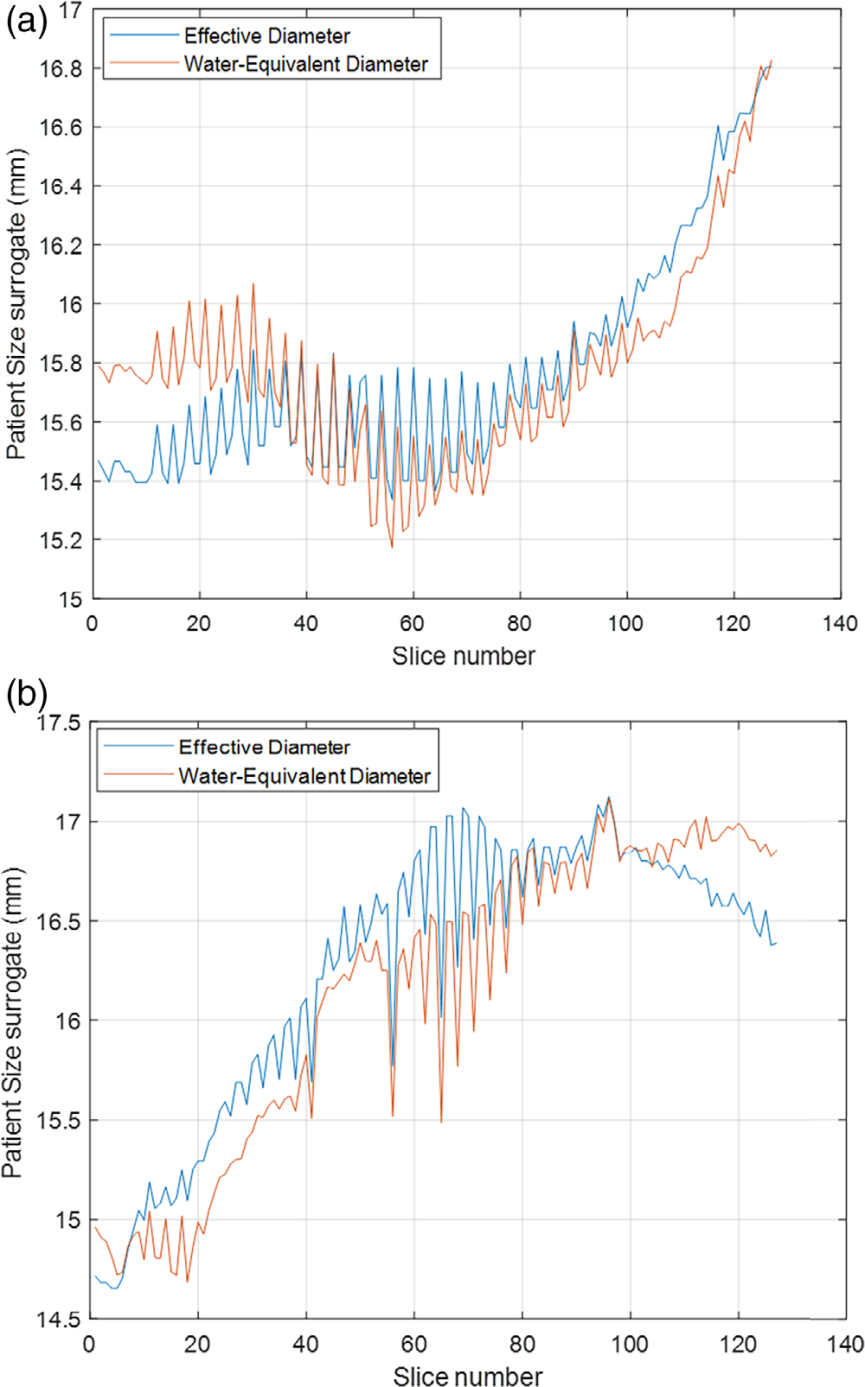
(a and b) shows two examples of the water‐equivalent diameter and effective diameter as a function of slice location for CT localizers on a patient scan.

Figure 5a,b demonstrates the WED and the effective diameter on a per slice basis using the CT localizer over the scan length. Vendors use modulation based on geometric size surrogates (LAT + AP), but having an accurate description of WED would improve the quality of the automatic exposure control, thereby reducing dose to the patient, particularly in the chest region where the density of lung is lower compared to PMMA in a CTDIvol phantom.

The advantage of using a calibration method is the linear relationship can be generated once for CT axial and CT localizers using phantoms that are readily available. It is possible to use patient data to generate calibration curves rather than phantoms. This option may be preferred as deep learning becomes more prevalent in diagnostic imaging. Vendors that are using CT localizers to determine CTDI_vol_ prior to the scan are using the geometric patient size surrogates, such as effective diameter, lateral and AP dimensions. The caveat with geometric size surrogates is they do not include the attenuation properties of the patient and that is demonstrated in Figure 3a,b.

This study explored how the lifetime achievable risk (LAR) for chest and abdomen correlates with the SSDE and CTDIvol. Both the SSDE and CTDIvol do not correlate well with LAR. However, the risk sensitivity improved with using SSDE calculated from the WED. The NDC has the best correlation with LAR for chest (*R* ^2^ = 0.18) and abdomenpelvis (*R* ^2^ = 0.19). For SSDE calculated for chest, there was a noticeable improvement in correlation for SSDE calculated from WED (*R* ^2^ = 0.051) from SSDE calculated from DE (*R* ^2^ = 0.035). The difference between the SSDE calculations is that WED incorporates the patient attenuation information, whereas the DE only provides geometric information and does not consider that the chest will have a lower attenuation due to the lungs. Also, for the chest, the RSI was higher for SSDE calculated from WED (RSI = 0.089) compared to SSDE calculated from ED (RSI = 0.071). For the abdomenpelvis region, the correlation did not change significantly between LAR and SSDE calculated from WED (*R* ^2^ = 0.012) and ED (*R* ^2^ = 0.011). The abdomen pelvis region has tissue properties similar to water, so the WED and ED will be similar. Overall, the SSDE did not correlated as well with LAR as did the CTDIvol. This is perhaps because the organ doses used to calculate LAR come from the CTDIvol. The NDC on its own has a better correlation than CTDIvol. It appears that the SSDE loses its sensitivity to radiation because it is the product of NDC and CTDIvol. This is consistent with a previous study that showed that the SSDE and CTDIvol did not correlate well with LAR, however they noted that the SSDE lost radiation risk sensitivity. It was unclear if that study used WED or ED to calculate SSDE.

Estimating WED from CT localizer radiographs would allow for WED to be included into data‐driven clinical workflows such as size adaptive protocol selection using diagnostic reference ranges which provide a minimum estimated patient dose.^17^, ^18^ The additional benefits of using CT localizers include reduced data overhead if CT axial images are not stored and possible limitations such as couch removal^10^, ^19^ related to CT axial calculation.

## 5 CONCLUSION

This study validates a calibration method showing an accurate estimate of water‐equivalent diameter (WED) from CT localizers for patient scans. The CT localizer pixel values are not absolute therefore the calibration method must be performed for different kVps on every scanner. The AAPM TG Report 220 states that using the WED is the preferred method to calculating size‐specific dose estimates, therefore it would be preferred over the geometric size surrogates for automatic exposure control settings. This study shows that the normalized dose coefficient can be determined within 20% as recommended by the AAPM TG Report 220. This study also demonstrates how the radiation sensitivity improves for SSDE using an attenuation‐based patient size surrogate.

## AUTHOR CONTRIBUTIONS

The corresponding author is responsible for data gathering, interpretation, and analysis. The corresponding and second author are responsible for written materials. The second author is responsible for data gathering.

## CONFLICT OF INTEREST STATEMENT

The authors declare no conflicts of interest.

## Data Availability

Data openly available in a public repository that issues datasets with DOIs.

## References

[1] McNitt‐Gray MF . AAPM/RSNA physics tutorial for residents: topics in CT. Radiation dose in CT. Radiographics. 2002;22(6):1541‐1553. 12432127 10.1148/rg.226025128

[2] Boone JM . The trouble with CTDI100. Med Phys. 2007;34(4):1364‐1371. 17500467 10.1118/1.2713240

[3] McCollough C , Cody D , Edyvean S , Diagnostic Imaging Council CT Committee and others . AAPM report no. 96 The Measurement, Reporting, and Management of Radiation Dose in CT. American Association of Physicists in Medicine; 2008.

[4] Bauhs JA , Vrieze TJ , Primak AN , Bruesewitz MR , McCollough CH . CT dosimetry: comparison of measurement techniques and devices 1. Radiographics. 2008;28(1):245‐253. 18203941 10.1148/rg.281075024

[5] Huda W , Scalzetti EM , Roskopf M . Effective doses to patients undergoing thoracic computed tomography examinations. Med Phys. 2000;27:838‐844. 10841385 10.1118/1.598949

[6] Menke J . Comparison of different body size parameters for individual dose adaptation in body CT of adults. Radiology. 2005;236:565‐571. 16040914 10.1148/radiol.2362041327

[7] Toth T , Ge Z , Daly MP . The influence of patient centering on CT dose and image noise. Med Phys. 2007;34:3093‐3101. 17822016 10.1118/1.2748113

[8] Wang J , Christner JA , Duan X , Leng S , Yu L , McCollough CH . Attenuation‐based determination of patient size for the purpose of size specific dose estimation in CT: part II. Implementation on abdomen and thorax phantoms using cross sectional CT images and scanned projection tadiograph images. Med Phys. 2012;39:6772. 23127071 10.1118/1.4757586

[9] Burton CS , Szczykutowicz TP . Evaluation of AAPM reports 204 and 220: estimation of effective diameter, water‐equivalent diameter, and ellipticity ratios for chest, abdomen, pelvis, and head CT scans. J Appl Clin Med Phys. 2018;19(1):228‐238. 29178549 10.1002/acm2.12223 PMC5768014

[10] Burton CS . Method of determining geometric patient size surrogates using localizer images in CT. J Appl Clin Med Phys. 2020;21(3):178‐183. 10.1002/acm2.12814 PMC7075380 31990136

[11] Zhang D , Mihai G , Barbaras LG , Brook OR , Palmer MR . A new method for CT dose estimation by determining patient water equivalent diameter from localizer radiographs: geometric transformation and calibration methods using readily available phantoms. Med Phys. 2018;45(7):3371‐3378. 29746705 10.1002/mp.12954

[12] (2006) Health risks from exposure to low levels of ionizing radiation: BEIR VII phase 2. National Academies Press. 25077203

[13] Li X , Samei E , Segars WP , et al. Patient‐specific radiation dose and cancer risk estimation in CT: part II. Application to patients. Med Phys. 2011;38:408‐419. 21361209 10.1118/1.3515864 PMC3021563

[14] Burton CS , Malkus A , Ranallo F , Szczykutowicz TP . Model‐based magnification/minification correction of patient size surrogates extracted from CT localizers. Med Phys. 2019;46(1):165‐172. 30372531 10.1002/mp.13251

[15] Ria F , Fu W , Hoye J , Segars WP , Kapadia AJ , Samei E , Comparison of 12 surrogates to characterize CT radiation risk across a clinical population. Eur Radiol. 2021;31:7022‐7030. 33624163 10.1007/s00330-021-07753-9 PMC11229091

[16] Lee C , Kim KP , Bolch WE , Moroz BE , Folio L . NCICT: a computational solution to estimate organ doses for pediatric and adult patients undergoing CT scans. J Radiol Prot. 2015;35(4):891‐909.2660999510.1088/0952-4746/35/4/891

[17] Boone JohnM . Reply to “Comment on the ‘Report of AAPM TG 204: size‐specific dose estimates (SSDE) in pediatric and adult body CT examinations’”[AAPM Report 204, 2011]. Med Phys. 2012;39(7):4615. 28516563 10.1118/1.4725757 PMC3412437

[18] McCollough C , Bakalyar DM , Bostani M , et al. Use of water equivalent diameter for calculating patient size and size‐specific dose estimates (SSDE) in CT: the report of AAPM task group 220. AAPM Report. 2014;2014:6. 27546949 PMC4991550

[19] McCollough C , Leng S , Yu L , et al. Size‐specific dose estimates (SSDE) in pediatric and adult body CT examination. AAPM Report. American Association of Physicists in Medicine; 2016.

